# Miniaturized Pyrotechnic Systems Meet the Performance Needs While Limiting the Environmental Impact

**DOI:** 10.3390/mi13030376

**Published:** 2022-02-26

**Authors:** Carole Rossi, Ruiqi Shen

**Affiliations:** 1 LAAS-CNRS, University of Toulouse, 7 Avenue du Colonel Roche, 31400 Toulouse, France; 2 School of Chemical Engineering, Nanjing University of Science and Technology, Nanjing 210096, China; rqshen@njust.edu.cn

Pyrotechnic systems, also termed pyrotechnics, refer to a broad family of sophisticated single-use devices that are able to produce heat, light, smoke, sound, motion, and/or a combination of these thanks to the reaction of an energetic material (primary and secondary explosives, powders/propellants, and other pyrotechnic substances). Most pyrotechnics utilize a simple hot wire or bridgewire to initiate the energetic material reaction and are used to perform a large variety of functions in large equipment, such as release, cutting, pressurization, valving, ignition, switching, and other mechanical works. Their applications are expanding in defense, civil engineering, demolition, fireworks, automotive, and space industries and are enjoying good safety records.

Two decades ago, the concept of micro-pyrotechnics emerged with the idea of reducing the manufacturing cost by applying collective microelectronic processing w to lower the ignition energy costs while improving both vulnerability and safety requirements by replacing the simple hot wire with a sophisticated arm and fire electronic unit.

At this stage, it was only very early thinking about how to manufacture new performing pyrotechnically actuated microsystems. The concept was considered only “technically feasible”, and researchers hoped to provide a cogent view that a new era in pyrotechnics was upon us, wherein the micro-nanotechnologies and simulation would allow entirely new capabilities to be developed with exciting advancements in the fields of propulsion, actuation, and thermics. Consequently, an active research effort was born internationally on both the design and elaboration of new nano-energetical materials (nano-energetics [1,2]) and the demonstration of new functionalities such as micro-actuators [3,4,5], micro-thrusters [6,7,8,9,10,11], tunable initiation of secondary explosives [12,13,14,15], joining, brazing, and sealing [16].

Two decades later, in 2022, advancements in energetic materials and micro-pyrotechnics are considerable, and the opportunity for new capabilities for industries built on micro-pyrotechnics is upon us. 

This Special Issue illustrates some of the works of the groups engaged in this research field. The first paper by Pouchairet and co-workers [17] presents the development of a miniaturized smart infrared (IR) electronically controllable flare combining a microinitiation stage that integrates low-energy addressable pyroMEMS (pyrotechnical micro-electromechanical systems) with a structured IR pyrotechnical loaf. Miniaturization is a key point of this work, but the choice of environmentally benign materials and technologies is also significant. Another series of papers presents innovative research on energetic composites that can be integrated into miniaturized devices for initiation. Liu and co-workers [18] develop a new composite energetic film (Cu(N_3_)_2_) on a MEMS chip, which presents high reactivity with better safety. Yu and co-workers [19] present a new generic synthesis route for CoFe_2_O_4_/Al nanothermite films by integrating Al nanoparticles with CoFe_2_O_4_ nanowires. Interestingly, this method is totally compatible with MEMS technologies and can be applied to diverse thermite systems, such as MnCo_2_O_4_ and NiCo_2_O_4_. He and co-workers [20] developed an explosive ink that can be printed layer by layer, each single layer being ~10 μm. The critical detonation size of the sample can reach 1 mm × 0.01 mm or less, and the detonation velocity can achieve 8686 m·s^−1^, which exhibits excellent micro-scale detonation ability. Finally, the two last papers consider miniaturized initiation devices. Wang and co-workers [21] present exploding foil microinitiators completely fabricated by MEMS-based engineering, which can be triggered by Metal-Oxide Semiconductor-Controlled Thyristor. Additionally, Lei and co-workers [22] integrate Cu/Ni Multilayer Exploding Foil on MEMS chips by Magnetron Sputtering and Electroplating.

It is only a snapshot of the current state of this research field, but these papers may encourage readers to investigate further. One important requirement for future miniaturized pyrotechnical systems is meeting the performance needs while maintaining a low cost and minimizing the environmental impact. Although it seems difficult to minimize the impact of a device that involves the combustion of fuel with the subsequent emission of gaseous and unburnt products, the choice of ingredients, such as environmentally friendly thermite, and the design of the system can reduce that impact significantly. 
